# Spatial Growth Factor Delivery for 3D Bioprinting of Vascularized Bone with Adipose-Derived Stem/Stromal Cells as a Single Cell Source

**DOI:** 10.1021/acsbiomaterials.3c01222

**Published:** 2024-02-28

**Authors:** Meric Goker, Utku Serhat Derici, Seyda Gokyer, Mehmet Goktug Parmaksiz, Burak Kaya, Alp Can, Pinar Yilgor

**Affiliations:** † Department of Biomedical Engineering, Ankara University Faculty of Engineering, Ankara 06830, Turkey; ‡ Department of Anatomy and Regenerative Medicine, Royal College of Surgeons in Ireland, Dublin D02 YN77, Ireland; § Department of Plastic, Reconstructive and Aesthetic Surgery, Ankara University Faculty of Medicine, Ankara 06620, Turkey; ∥ Ankara University Medical Design Research and Application Center, MEDITAM, Ankara 06520, Turkey; ⊥ Department of Histology and Embryology, Ankara University Faculty of Medicine, Ankara 06230, Turkey

**Keywords:** vascularized bone, bioprinting, controlled release, microencapsulation, perfusion culture, bioreactor

## Abstract

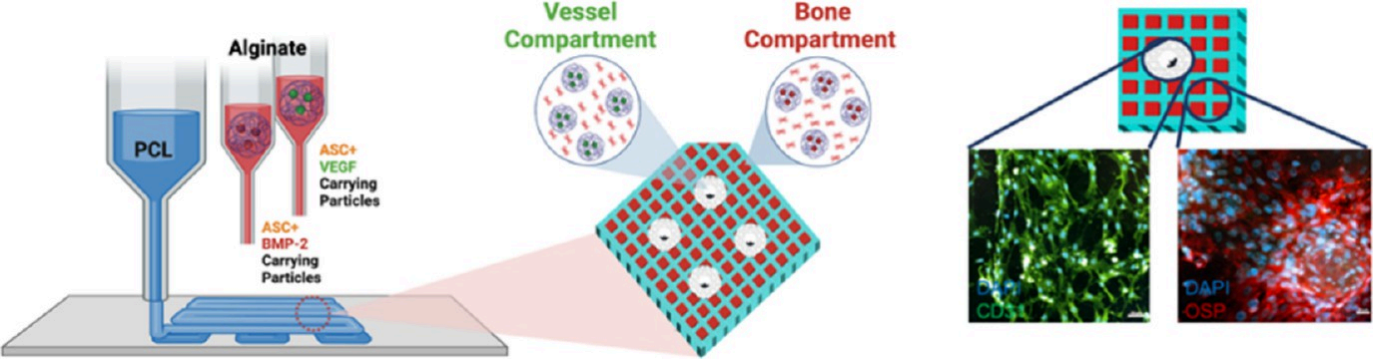

Encapsulating multiple growth factors within a scaffold enhances the regenerative capacity of engineered bone grafts through their localization and controls the spatiotemporal release profile. In this study, we bioprinted hybrid bone grafts with an inherent built-in controlled growth factor delivery system, which would contribute to vascularized bone formation using a single stem cell source, human adipose-derived stem/stromal cells (ASCs) in vitro. The strategy was to provide precise control over the ASC-derived osteogenesis and angiogenesis at certain regions of the graft through the activity of spatially positioned microencapsulated BMP-2 and VEGF within the osteogenic and angiogenic bioink during bioprinting. The 3D-bioprinted vascularized bone grafts were cultured in a perfusion bioreactor. Results proved localized expression of osteopontin and CD31 by the ASCs, which was made possible through the localized delivery activity of the built-in delivery system. In conclusion, this approach provided a methodology for generating off-the-shelf constructs for vascularized bone regeneration and has the potential to enable single-step, in situ bioprinting procedures for creating vascularized bone implants when applied to bone defects.

## 1 Introduction

There are numerous regulatory factors involved in the bone healing process along with the presence of a plethora of cells ranging from osteocytes, osteoclasts, osteoblasts, and their osteoblastic precursors such as mesenchymal stem cells (MSCs).^1−3^ However, the presence of these cells alone is insufficient to initiate the bone healing cascade. Correct signaling sequences of both osteoconductive and osteoinductive factors including bone morphogenic proteins (BMPs) are essential.^4,5^ Equally important in this healing cascade is the coactivity of angiogenic cells and their well-orchestrated physiological process known as angiogenesis.^6,7^ It was discovered a century ago that the bone tissue possesses a remarkable network of highly vascularized blood vessels, extending through its osteons, Haversian/Volkmann’s canals in the cortical section, and penetrating into the medullary-positioned cancellous section.^8^ Clinical evidence has shown that the absence of these blood vessel networks within bone implants can result with major problems, such as necrosis in the center of the large grafts.^9−11^ Moreover, the lack of a bone vessel network leads to poor graft viability and nonuniform osteointegration, both of which can ultimately lead to graft failure in the postoperative phase.^5,8,12,13^ One recent approach to accelerate bone restoration involves enhancing full graft integration by incorporating deep functional vasculatures within the bone graft system.^14−16^

Controlled release of bioactive agents from scaffolds is a critical research focus in regenerative medicine because modulating cellular activities plays a significant role in the regeneration process.^17−19^ Since the natural bone formation process is fairly complex and involves multiple growth factors released in different regions of the bone tissue, delivering multiple factors to the forming tissue microenvironment is essential.^20^ Scaffold-based applications of bone tissue engineering have introduced innovative concepts over the last few decades to construct multifaceted tissue grafts capable of fulfilling all necessary functions.^21^ Nonetheless, conventional scaffold fabrication methodologies face certain limitations such as the challenge of fabricating highly ordered, porous, and complex architectures.^22^ Ever since the introduction of 3D printing technology, research focus has shifted in this direction due to its innovative and groundbreaking capabilities in customizing manufactured products compared to standard manufacturing practices.^23^ This state-of-art technology enables researchers to fabricate intricate geometries with high precision, accuracy, and most importantly reproducibility.^24^ Accordingly, bone tissue engineering utilizes 3D printing of cell-laden bioinks, known as bioprinting, to harness its versatile advantages.^25,26^ This additive manufacturing technology allows precise control over all aforementioned properties, as well as controlled spatial distribution, and the deposition of multiple cell-laden biomaterials in a layer-by-layer manner.^27^ For these reasons, in this study, we aimed to utilize bioprinting technology to fabricate sophisticated scaffolds with spatiotemporal inductive cues by incorporating human adipose-derived stem cells and stromal cells (ASCs) into predetermined locations.

**Figure 1:**
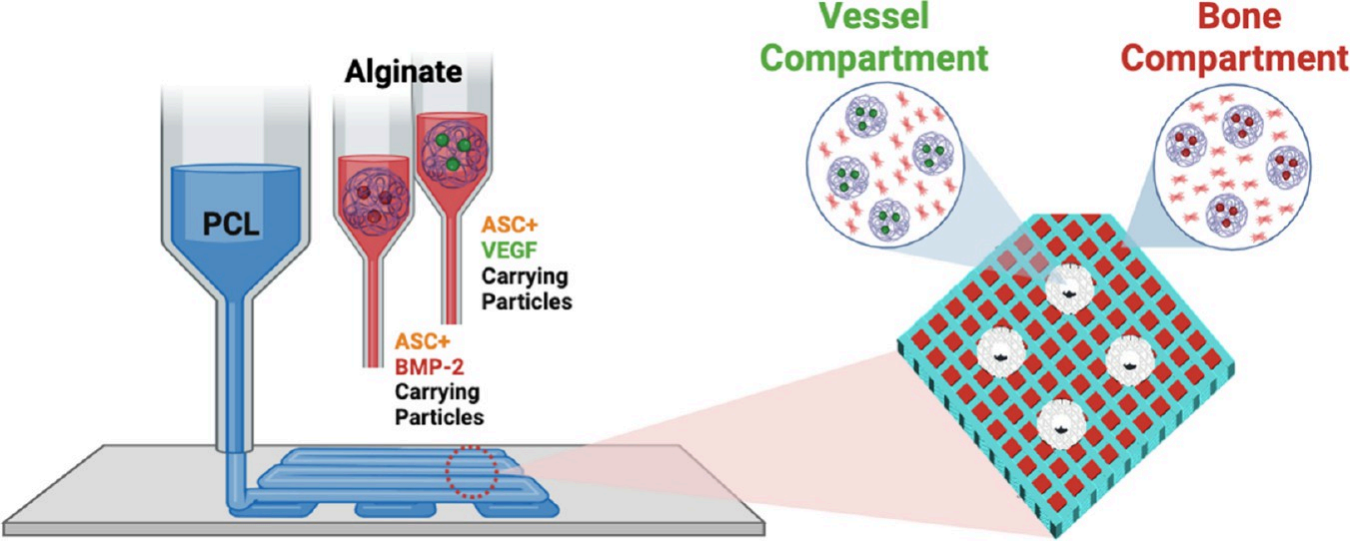
Schematic representation of the hierarchical and spatial organization followed for 3D bioprinting of the vascularized bone graft. Three different print heads were used for the biofabrication. The first print head was used to 3D print PCL. The second print head included *osteogenic bioink* (ASCs and BMP-2-loaded particles within alginate). The third print head was used to bioprint *angiogenic bioink* (ASCs and VEGF-loaded particles within alginate). The magnified figure on the right-hand side represents the structure of the bioprinted vascularized bone graft with distinct regions including (i) PCL (blue lines), (ii) osteogenic bioink (bone compartment, red squares), and (iii) angiogenic bioink (vessel compartment, white circles).

ASCs, a heterogeneous source of precursor cells, possess MSC properties and osteogenic tissue formation potential upon osteoinduction (such as BMPs). Additionally, they include endothelial cells capable of inducing *de novo* vessel formation within a synthetic graft through stimulation with the vascular endothelial growth factor (VEGF).^28^ In this work, BMP-2 and VEGF proteins were encapsulated in polymeric microparticles and placed at predetermined locations within the graft during bioprinting. Polycaprolactone (PCL) was coprinted with ASC-laden bioink, and growth factor-loaded microparticles were supplemented within the alginate-based bioink. The native structure of the vascularized bone tissue was practically mimicked by means of a unique architectural design. Figure 1 illustrates the methodology followed in this approach in order to produce a bioprinted vascularized bone structure.

## 2 Methods

### 2.1 Microencapsulation of BMP-2 and VEGF

PCL microcapsules were produced following a previously established method.^29^ Briefly, PCL was dissolved in methylene chloride at varying concentrations. Aqueous solutions of BMP-2 and VEGF were added and sonicated for 15 s at 50 Hz. The resulting emulsion was then introduced to a 4% (w/v) poly(vinyl alcohol) (PVA) solution and underwent further sonication under the same conditions. The double emulsion was homogenized in 0.3% (w/v) PVA solution, and the solvent was evaporated under continuous stirring overnight. The microcapsules were subsequently rinsed with Tris–HCl (10 mM, pH: 7.4) and lyophilized for 24 h. The morphology of the microcapsules was assessed using scanning electron microscopy (SEM, Quanta 400F Field Emission SEM) after sputtering with gold.

### 2.2 Assessment of Encapsulation Efficiency and Release Kinetics

To evaluate release kinetics and encapsulation efficiency, bovine serum albumin (BSA) was employed as a model protein since it has a molecular weight (66 kDa) comparable to those of BMP-2 and VEGF (ca. 40 kDa). 5 mg of microparticles was suspended in 1 mL of phosphate-buffered saline (PBS) solution (pH 7.4) and incubated at 37 °C for 21 days. At various time points (days 1, 3, 7, 14, and 21), the samples were subjected to centrifugation and the supernatants were collected. The particles were subsequently resuspended in fresh 1 mL of PBS. Quantification of BSA was performed using the Bradford assay (Coomassie Plus Bradford Assay, Pierce) according to the manufacturer’s instructions. To determine the encapsulation efficiency, microparticles were disrupted in methylene chloride and the protein content was extracted with distilled water prior to content quantification. Release kinetics were assessed from the free microparticles and loaded particles embedded within the alginate bioink. A similar procedure was applied by suspending microparticle-loaded 3D-printed scaffolds in PBS.

### 2.3 Adipose-Derived Stem/Stromal Cell Isolation and Culture

ASCs were isolated from human subcutaneous adipose lipoaspirates obtained from Ankara University Faculty of Medicine, Department of Reconstructive and Plastic Surgery, in accordance with Clinical Research Ethical Committee approval (#13-25-15). The cell isolation protocol was followed as described previously.^30^ In brief, lipoaspirate was enzymatically digested by using 0.1% collagenase type I, 1% BSA, 2 mM CaCl_2_ in PBS for 1 h at 37 °C. Samples were then centrifuged to isolate the stromal vascular fraction (SVF). Subsequently, the SVF pellet was plated onto tissue culture dishes to obtain the plastic-adherent population (P0). Vials containing P0 were cryopreserved and stored until use. ASCs were then thawed in expansion medium (Dulbecco’s modified Eagle's medium (DMEM) supplemented with 10% fetal bovine serum (FBS), 1% penicillin/streptomycin (P/S), and 1 ng/mL FGF-2) under standard culture conditions (37 °C and 5% CO_2_). P3 ASCs were employed for all experiments conducted in this study.

### 2.4 Bioprinting of Vascularized Bone Scaffolds

PCL (Perstorp AB, Sweden) (*M*_w_ = 37000 g/mol) was melted in the high-temperature print head of the 3D Bioplotter (EnvisionTEC, Germany) at 130 °C. The molten PCL was extruded through a syringe (460 μm) at 3.5 bar and 3D printed on a 37 °C glass surface in the form of filaments. Other print heads were loaded with 2% (w/v) sterile alginate solution (loaded with 1.25 × 10^6^ ASCs/mL and GF-laden microparticles) and was coprinted as filaments. Cross-linking of the bioink was achieved using a sterile 0.5 M CaCl_2_ solution.

To optimize and characterize the manufacturing process, cubic scaffolds with dimensions of 10 × 10 × 15 mm were printed. Subsequently, to establish the feasibility of our concept, anatomically shaped scaffolds were designed and 3D printed, aiming to craft custom-made vascularized bone grafts using a single-cell source (ASCs). Patient DICOM data from an MRI scan was obtained from the Ankara University Department of Plastic, Aesthetic and Reconstructive Surgery Clinic (Approval No.: 13-25-15). The 3D model generation and implant design were performed using Mimics software (Materialise). After radiological image segmentation, 3D image creation (surface rendering) was carried out, followed by 3D implant design using the MIMICS software’s “Materialise-3-matic” module. 3D models produced for patients from DICOM images were obtained as .stl files and further used in the 3D printing.

Circular canals were designed and incorporated into the hybrid scaffolds to localize neovascularization triggered by VEGF release (vessel configuration was optimized as described below). Print optimization was conducted to enhance reproducibility, repeatability, and rigidity by adjusting parameters such as the fiber spacing and fiber arrangement. Additionally, the linear speed of the print head, loading density, print pressure, and temperature were also optimized for the ideal construction of the inner structure.

For the preparation of the bioink, alginate powder, BMP-2, and VEGF-laden PCL nanocapsules were sterilized under UV light for 30 min. BMP-2 (40 ng/mL) and VEGF (10 ng/mL) were loaded per scaffold, with doses determined previously.^4,31,32^ ASCs collected from P3 were resuspended in 4% (w/v) sterile alginate solution, along with the microcapsules, at a concentration of 1.25 × 10^6^ cells/mL for both osteogenic and angiogenic bioinks. 3D grafts were incubated in 0.5 M CaCl_2_ solution for 5 min to cross-link the bioinks prior to culture (static or perfusion) for 21 days.

### 2.5 Optimization of Vessel Configuration in 3D-Bioprinted Scaffolds

The 3D printing of the vascularized bone structure was carried out by creating distinct vessel and bone compartments within the graft structure, as depicted in Figure 1. The inclusion of each of these compartments facilitated the accommodation of both osteogenic and angiogenic bioinks. In order to optimize the spatial arborization of the vessels in the bone matrix, several 3D models were developed, drawing inspiration from the Haversian canal microarchitecture found in native tissue. As a result, seven different types of vessel and bone structure placements were designed and fabricated with the 3D Bioplotter. The effect of vessel placement on the mechanical strength of the hybrid bone graft was evaluated through compression testing (as described below). Straight cylindrical and three-spin (slalom) vessel structures (one slalom, two slalom, and four slalom configurations) were printed to investigate the impact of 3D vessel geometry on the mechanical strength of the vascularized bone graft.

### 2.6 Uniaxial Compression Testing

Uniaxial compression testing was conducted to assess the mechanical strength of the grafts by using a Universal Testing Machine (Shimadzu AGS-X, Japan). A 50 kN load cell was employed with a linear compression speed set at 200 μm/min. The amount of stroke applied was adjusted in accordance with the specimen’s thickness. Stress–strain graphs were utilized to derive mechanical properties, including Young’s modulus and ultimate compressive strength.

### 2.7 FTIR Analysis

Characterization of PCL and PCL microparticle-loaded alginate bioink structures was carried out using Fourier transform infrared (FTIR) spectroscopy (Jasco FT/IR Spectrometer 4600, Japan). The transmittance was calculated, and the FTIR spectra were recorded by performing 128 scans with a resolution of 4 cm^–1^. Samples were categorized as follows: (1) PCL 3D scaffold, (2) growth factor-laden PCL microcapsules, (3) 3D alginate scaffold, (4) PCL microcapsule-loaded 3D alginate scaffold. FTIR analyses were processed using the KnowItAll Informatics System (Wiley, 2023), and the spectra between 500 and 1450 cm^–1^ were selected to enhance the clarity of the analysis, with a focus on the regions containing the remaining functional groups. Subsequently, the analysis was completed with the regions using this refined spectral range.

The bioprinted vascularized bone scaffolds were subjected to culture under both static cell culture conditions and a perfusion bioreactor system. In static culture, the scaffolds were placed in 12-well plates and submerged in DMEM-based high-glucose growth media, supplemented with 10% FBS, 1% P/S, and 1 ng/mL FGF-2. The medium was refreshed every other day throughout a 21-day culture period. Osteogenic induction was made possible by the DMEM-based low-glucose media, supplemented with 10% FBS, 1% P/S, 100 nM dexamethasone, 50 μM ascorbic acid, and 10 mM glycerol 2-phosphate.

Bioprinted vascularized bone scaffolds were cultured in a perfusion bioreactor system (3D CulturePro Electroforce, TA Instruments) for 21 days to ensure the effective nutrition and homogeneously distributed cell survival in the 3D matrix. 100 mL of growth medium was perfused at a rate of 60 rpm.

### 2.9 Determination of Viable Cell Number and Morphology

The number of living cells within the grafts was determined using the Alamar Blue assay (US Biological) at days 1, 7, 14, and 21. Shortly, samples were incubated under standard cell culture conditions for 1 h with Alamar Blue solution in DMEM without phenol red (10% v/v). Following the incubation period, 200 μL of the test solution was transferred to 96-well plates and optical density was measured on a microplate reader.

Both living and dead cells within the 3D grafts were stained with the LIVE/DEAD Viability/Cytotoxicity kit (Invitrogen) and subsequently imaged under confocal scanning laser microscopy (CLSM, Zeiss-Germany). The morphology of the cells within the scaffolds was observed with SEM (Quanta 400F Field Emission SEM). Scaffolds were fixed within 3.7% (v/v) glutaraldehyde, washed in 0.1 M Sorenson buffer, dehydrated in a series of ethanol, dried in hexamethyldisilazane (HMDS), and finally coated with Au–Pd (15 nm). Observations were carried out at 20 kV, and images were recorded at low magnifications (50–500×).

### 2.10 Alizarin Red Staining

The osteogenic differentiation of ASCs was assessed through Alizarin red staining at various time points during culture period (days 7, 14, and 21). The samples were fixed in 3.7% paraformaldehyde (PFA) for 20 min, rinsed with distilled water, and then incubated with a 40 mM Alizarin Red solution at room temperature for 30 min. Any excess dye was removed by washing with distilled water until the rinse solution became clear. The samples were then left to air-dry overnight before examination under brightfield microscopy. Images were captured from the same areas of interest in all samples.

### 2.11 Alkaline Phosphatase Activity Assay

To assess ASC osteogenic differentiation, the activity of the osteoblast-specific enzyme alkaline phosphatase (ALP) was quantified. The samples were first rinsed with PBS and then preserved by freezing until the time of testing. Upon thawing, the frozen scaffolds were rinsed with PBS and subsequently lysed with a 1% Tris–Triton X-100 solution. Freeze–thaw cycles were performed in triplicate, followed by a 10 min sonication (30 s pulses with 30 s breaks). After centrifugation to remove cell debris and other components, 40 μL of the supernatant was diluted at a 1:2 ratio with ALP Assay buffer (Abcam, USA) and this mixture was then incubated in a 96-well culture dish, and 50 μL of the *p*-nitrophenyl phosphate (pNPP) substrate was added. The incubation occurred at 37 °C for 60 min. The reaction was halted by adding 40 μL of ALP Stop Solution (Abcam, USA), and the absorbance value was measured at 405 nm. The ALP enzyme activity was determined based on a standard curve prepared with known concentrations of pNPP.

### 2.12 Immunocytochemistry

The analyses of ASC-derived osteogenesis and angiogenesis within the grafts were conducted through immunocytochemistry. In brief, samples were fixed with 3.7% PFA on days 7, 14, and 21. Subsequently, the samples were rinsed with PBS and incubated in PBS 1× with 0.1% Tween 20, 1% BSA for 2 h at 4 °C. This step served the purpose of preventing nonspecific binding and permeabilizing the cell membranes. Following blocking and permeabilization, the samples were exposed to primary antibodies [mouse antiosteopontin (1:500) (Abcam ab8448) and rabbit anti-CD31 (1:200) (Abcam ab 9498)] that were diluted in PBS containing 1% BSA. This incubation took place at 4 °C overnight. Subsequently, the samples were treated with fluorochrome-conjugated secondary antibodies [Alexa Fluor 488-conjugated goat antimouse IgG (1:200) and Alexa Fluor 594-conjugated goat antirabbit IgG (1:200)] for 3 h at room temperature. To visualize cell nuclei, DAPI staining was carried out for 10 min at room temperature. Imaging was conducted by using CLSM (Zeiss).

### 2.13 Statistical Analysis

All quantitative results were presented as means ± standard deviation (*n* > 3). Statistical significance between groups (*p* < 0.05) was assessed through one-way analysis of variance (ANOVA), followed by Tukey’s post hoc tests.

## 3 Results

### 3.1 PCL Microcapsule Production and Assessment of Release Kinetics

**Figure 2:**
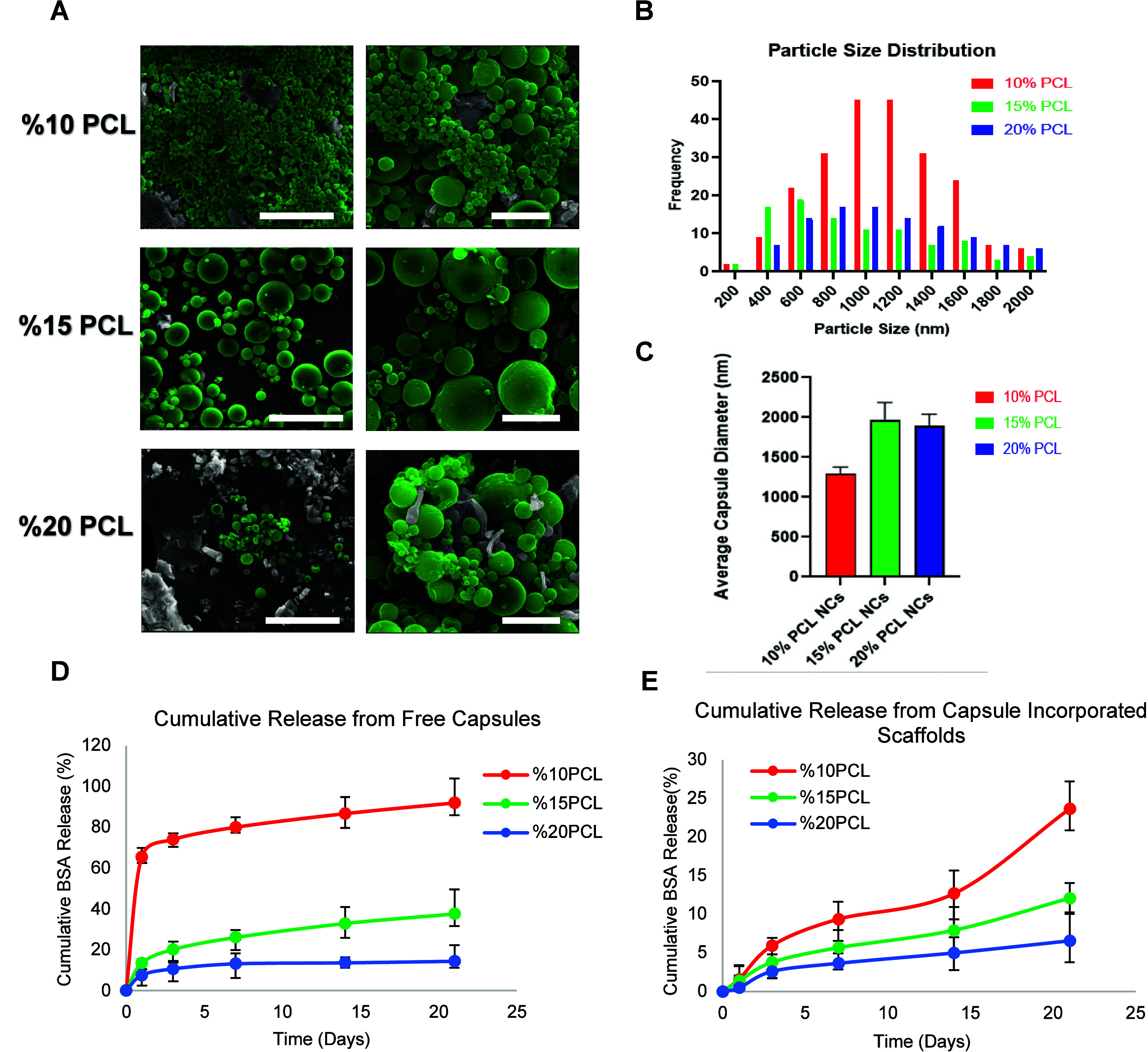
(A) SEM micrographs showing PCL particles at concentrations of 10, 15, and 20% w/v PCL particles, imaged at 2000× (left) and 8000× (right) magnifications. (B) Particle size distribution at different PCL concentrations. (C) Comparison of average capsule diameter among the groups. (D) BSA release from 10, 15, and 20% free PCL capsules. (E) BSA release from 10, 15, and 20% capsule-incorporated scaffolds at various time points (days 1, 3, 7, 14, 21).

Microparticles made from PCL were developed to encapsulate BMP-2 and VEGF. Prior to this, BSA was employed as a model protein to investigate the release kinetics and encapsulation efficiency. Different formulations of (5, 10, 15, and 20% (w/v)) PCL solutions were prepared. No capsular structure formation was observed with the use of 5% (w/v) PCL. PCL concentrations of 10% (w/v) and higher led to the formation of microcapsules, as shown in Figure 2A.

The frequency distribution of the microcapsules was evaluated by ImageJ software based on the SEM images at 8000× magnification. For this, spherical structures were detected and their diameters were measured by the straight-line tool of the software. GraphPad’s histogram analysis tool was used to sketch the frequency distribution of the microcapsules over the particle diameter, as shown in Figure 2B (*n* = 10). For all groups, particle size varied between 50 nm and 2 μm. For 10% (w/v) PCL capsules, the highest frequency of particle diameter was in between 800 and 1400 nm whereas smaller and larger particles were present in relatively less amount. Particles with smaller diameters below 400 nm were not observed under 20% (w/v) conditions. The majority of the capsule diameters in 15 and 20% (w/v) PCL groups were 1000 nm and above. The average diameter of 10% (w/v) condition was 1200 nm, while this average value goes up to 1900 nm for 15 and 20% w/v conditions (Figure 2C).

Encapsulation efficiency of the microcapsules was determined by using BSA as the model protein. Results indicated that encapsulation efficiency was not affected significantly by the particle diameter and/or the size distribution. Encapsulation efficiencies of 10, 15, and 20% (w/v) PCL microparticles were found to be 49.32% ± 2.50%, 57.06% ± 1.09%, and 60.18% ± 0.73%, respectively. Encapsulation efficiency increased with an increasing PCL concentration, although the increase was not statistically significant.

The impact of PCL concentration on the release kinetics, as previously mentioned with BSA as a model protein, was examined for both free particles and particle-laden bioink. The cumulative release profiles for both groups are shown in Figure 2D and E, respectively. It was observed that an increase of polymer concentration from 10 to 20% led to BSA release to decrease over time. Considering the thickened walls of capsules, increased polymer concentration has led to a more sustained release rate of the content. Furthermore, embedding capsules into the hydrogel matrix enabled BSA to have an even more sustained release profile over time.

### 3.2 Scaffold Mechanical Properties in Relation with the Vessel Configuration

**Figure 3:**
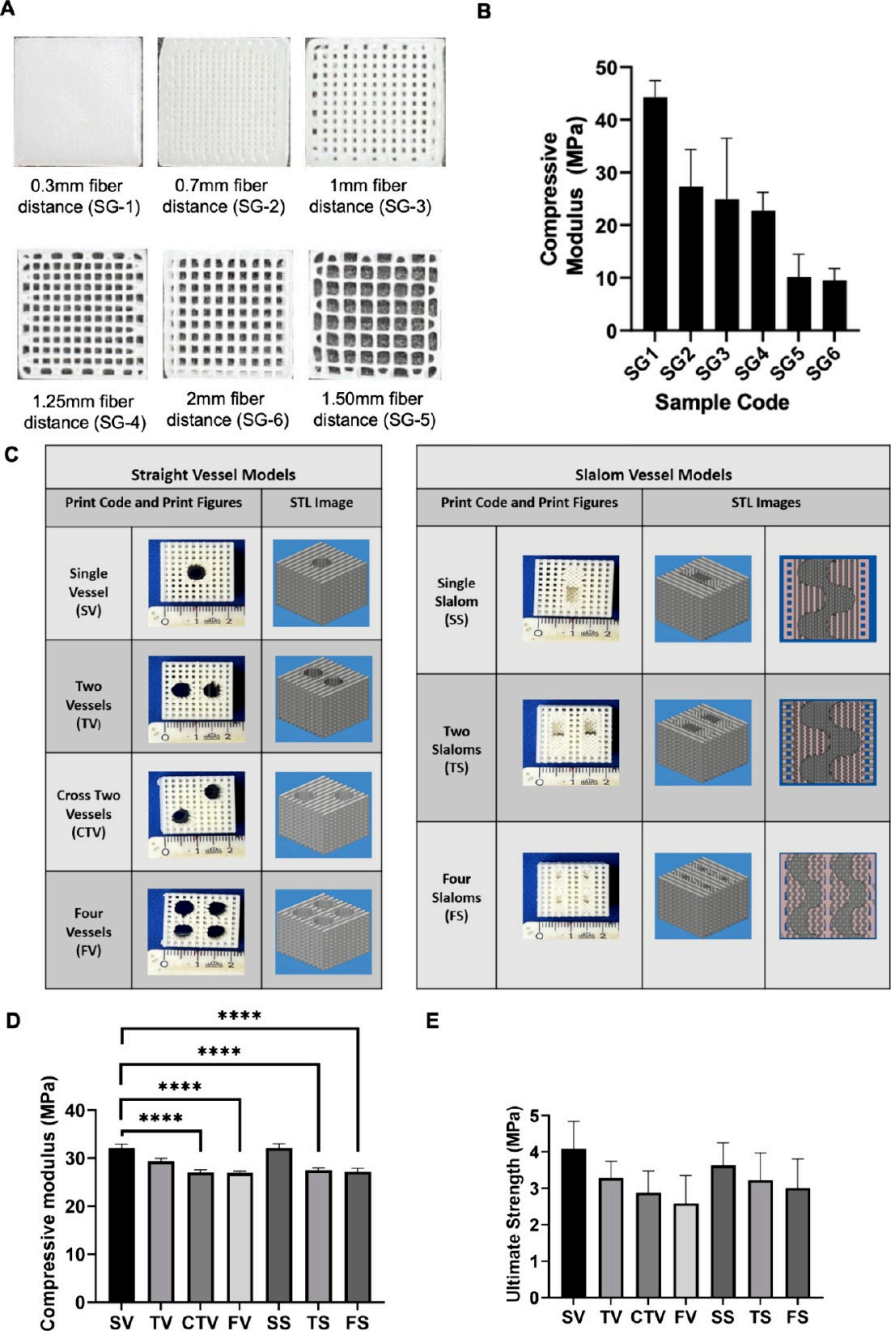
(A) Scaffold layouts with varying fiber distances and pore sizes. (B) Compressive modulus of canal-free square grids at 10% deformation. (C) CAD models of varying vessel configurations. (D) Compressive Young’s modulus. (E) Ultimate compressive strength of scaffolds with varying vessel configurations.

Vascularized bone scaffolds were produced with preformed vessel structures. The 3D vessel configuration was optimized for a higher compressive strength. For this, the effect of fiber distance during 3D printing was the first step. Figure 3A depicts the fiber distance and orientation, where Figure 3B shows the corresponding compressive modulus. Young moduli of the scaffolds decreased as the distance between the fibers increased, as expected, due to the reduced material presence. Despite the fact that Square Grid-1 (SG-1) exhibited the highest compressive modulus, it was not chosen due to its diminished porosity. Instead, the SG3 scaffold geometry, with a 1 mm fiber distance and appropriate porosity, was selected for further studies.

Several designs were developed in order to emulate the Haversian canal architecture in native bone tissue, with the goal of optimizing the vessel density across the graft and not hindering the mechanical stability and structural integrity. For this, a single canal was implemented in the center of the square grid. Subsequently, the canal layout was further optimized by introducing new canals into the configuration, as illustrated in Figure 3C. The results of the compression test are presented in Figure 3D,E. According to this, the single-vessel (SV) structure resulted in 30.23 ± 2.30 MPa compressive moduli and it gradually decreased as new canals were added (TV = 29.35 ± 3.30 MPa, FV = 26.94 ± 2.50 MPa). Diagonal placement of the vessels (CTV) further reduced the compressive modulus to 27.04 ± 3.98 MPa in comparison with TV (Figure 3D).

The 3D vessel configuration pattern was varied from straight lines to continuous slaloms in order to assess the effect of this biomimetic orientation on the mechanical properties of the scaffolds. This change resulted in slightly higher compressive moduli, as seen in the SS vs SV comparison. A similar trend has emerged when TS vs TV models and FS vs FV models were compared with each other. Furthermore, it was revealed that there were no statistically significant differences in ultimate compressive strength among different vessel configurations (Figure 3E).

### 3.3 Characterization of PCL Particles Embedded within the Alginate Bioink Matrix

**Figure 4:**
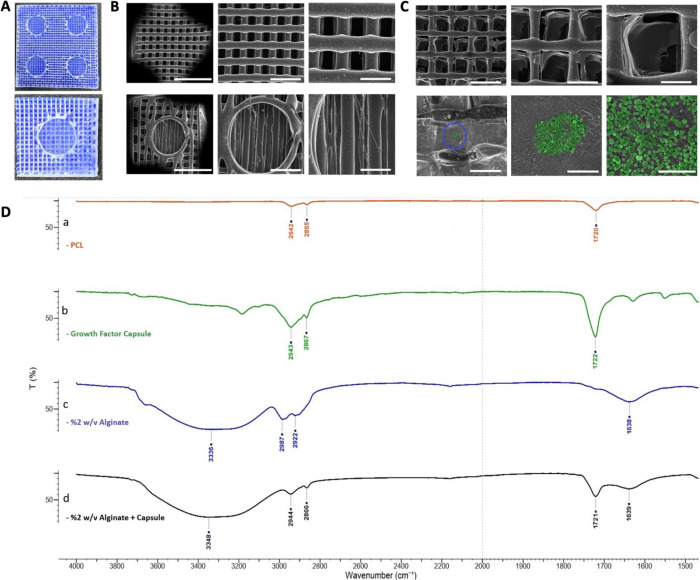
(A) Surface micrographs of 3D-printed PCL fibers. (B) SEM micrographs of alginate (capsule free)–PCL and (C) alginate (capsule-incorporated)–PCL scaffolds under 25×, 50×, and 100× magnification. (D) FTIR spectra of the PCL, PCL particle, alginate, and particle-incorporated alginate samples.

The morphology of 3D-printed PCL scaffolds without (Figure 4A,B) and with (Figure 4C) the alginate bioink matrix was investigated using SEM imaging. The presence of PCL particles within the alginate matrix was visualized.

FTIR spectroscopy was utilized to validate the presence of growth-factor-laden PCL particles within the alginate bioink matrix, and the corresponding spectra are presented in Figure 4D. Given that PCL was employed both in the scaffolding of the bone matrix and in the particle preparation, it was noted that the transmission values for the PCL filament and the capsule analysis results displayed similar patterns in the common bands. Specifically, the prominent IR peak observed at 1720–1722 cm^–1^ corresponds to vibrations occurring in the C=O band plane. The shared peaks at 2865–2867 and 2942–2943 cm^–1^ represent the strong symmetrical and antisymmetric stretch bands of the C–H bond, respectively.

The presence of alginate within the structure was confirmed by the corresponding peak. Consequently, the broad stretch band with the presence of the O–H hydrogen bond at the value of 3336–3348 cm^–1^ was evident in both spectra (alginate and alginate + PCL capsule). The peak at 1638–1639 cm^–1^ signified the asymmetric stretching vibration of the O–C–O carboxylate group in the alginate. Upon examination of the alginate–capsule group, it was evident that vibration bands at 2866 and 1721 cm^–1^ were present, originating from both the alginate solution (at 3348 and 1639 cm^–1^) and the PCL capsule.

### 3.4 Cell Viability and Proliferation Is Maintained within the Grafts under Static Culture Conditions

**Figure 5:**
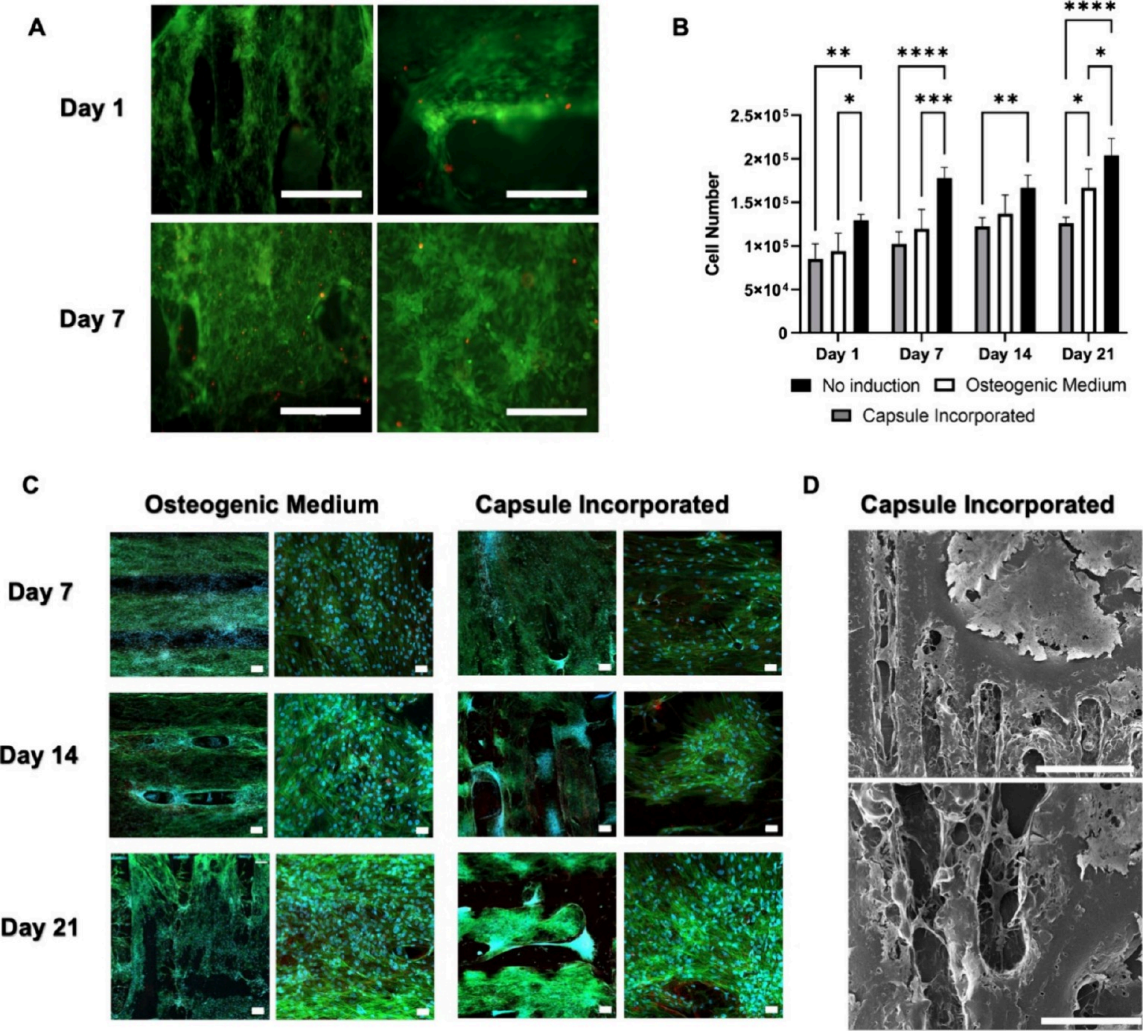
(A) Fluorescence images displaying live/dead staining of ASCs within the PCL scaffolds. Live cells were stained with calcein-AM (green), and ethidium homodimer-1 was used to stain dead cells (red). Scale bar = 400 μm. (B) Number of viable cells on scaffolds during static culture. Data are expressed as mean ± standard deviation of three different experiments. Significant differences are denoted with asterisks (*P* value: *<0.05, **<0.01, ***<0.001, ****<0.0001). (C) 3D Z-stack CLSM images comparing BMP-2 capsule-incorporated group vs control group from days 7 to 21 (scale bar = 200 μm,50 μm). Channels: green—phalloidin, blue—DAPI. (D) SEM micrographs of ASCs on the entire surface of the capsule-incorporated samples at day 21 in static culture (scale bar = upper image: 2 mm, below image: 500 μm).

All cell culture experiments were carried out with the following experimental groups: (I) no induction, (II) osteogenic medium, and (III) capsule incorporation (BMP-2 and VEGF release from scaffolds). Initially, cell viability was assessed qualitatively using the Live/Dead cell viability assay, and subsequently, metabolic activity was quantified through the Alamar Blue assay. The results revealed that cell viability was maintained in bioink despite the fact that cells were exposed to physical strain and high thermal exposure during the printing process and cells proliferated enough to cover the entire surface of the graft by day 7 (Figure 5A). The introduction of PCL microcapsules into the system did not significantly impact the number of viable cells, as shown in Figure 5B. However, the induction of osteogenic differentiation led to considerably less proliferation compared to the control group. Similarly, it was observed that addition of capsules containing BMP-2 and VEGF had no significant effect on cell viability. Examination of the groups over different time periods indicated a statistically significant increase in the number of viable cells between day 1 and day 21, signifying cell proliferation within the bioprinted structure.

The gradual increase in the number of viable cells from day 7 to day 21 aligned well with the cell viability results described above (Figure 5C). When encapsulated BMP-2 was integrated into the grafts, it was observed that cells had spread across the entire surface (Figure 5D), a finding consistent with the quantified number of viable cells within the grafts.

### 3.5 Osteogenesis and Vascularization Is Triggered Spatially by the Inherent Dual Growth Factor Delivery System

**Figure 6:**
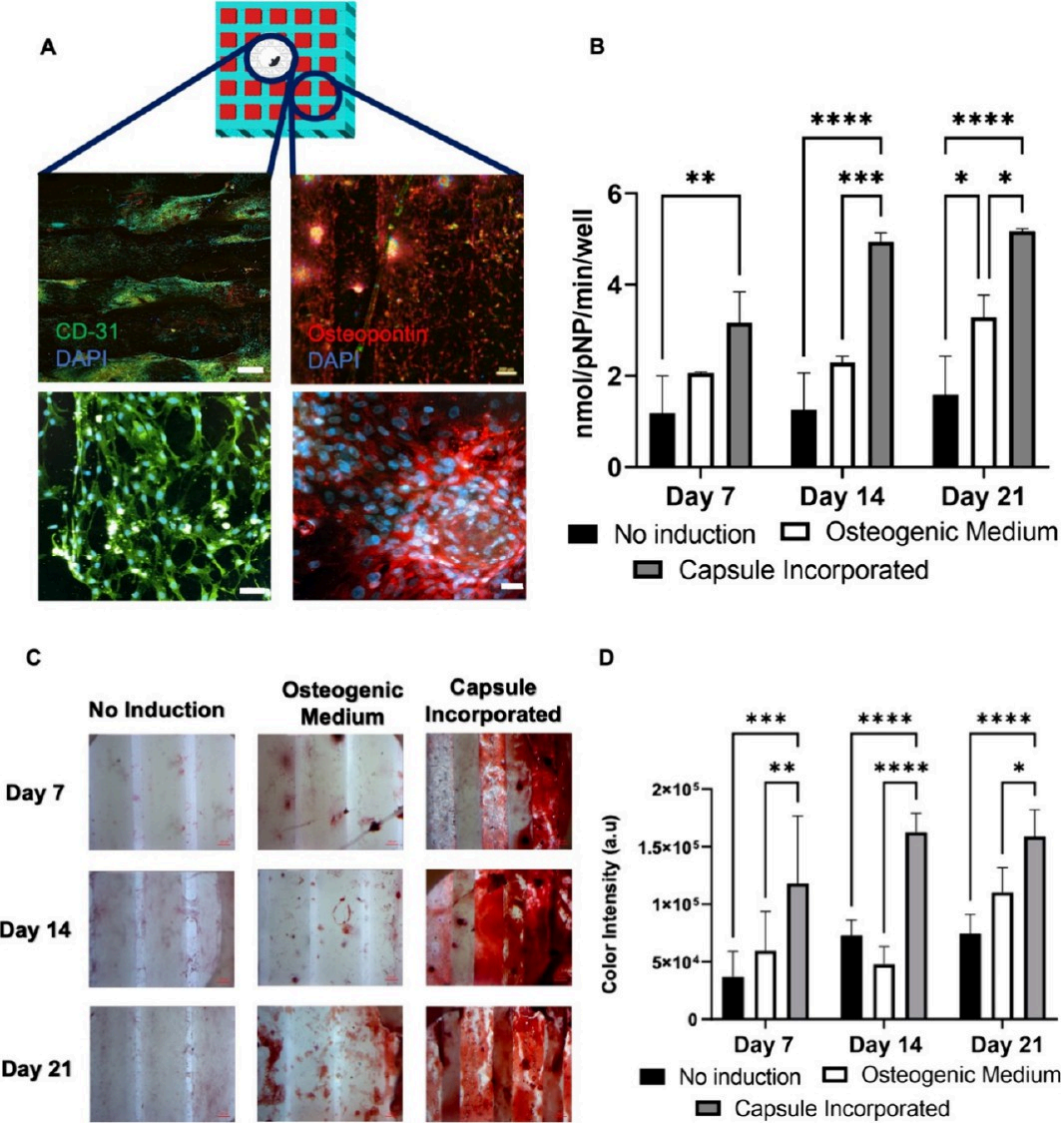
(A) Confocal laser scanning microscopy (CLSM) images illustrating dual growth factor delivery from the same scaffold on day 14. Scale bar = 200 μm (top) and 50 μm (bottom). Channels: red—osteopontin, green—CD-31, blue—DAPI. (B) Alkaline phosphatase (ALP) activity over time across all experimental groups. ALP enzyme activity reflects the amount of *p*-NP formed per minute. Data is expressed as mean ± standard deviation of three different experiments. Significant differences are marked with asterisks (*P* values *<0.05, **<0.01, ***<0.001, ****<0.0001). (C) Alizarin red staining of no induction, osteogenic medium, and capsule-incorporated groups for days 7, 14, and 21. Scale bar = 100 μm. (D) Quantification of average color intensity in alizarin red staining (*P* values: *<0.05, **<0.01, ***<0.001, ****<0.0001).

BMP-2 and VEGF were encapsulated within PCL particles to create the osteogenic and angiogenic bioinks combined with the ASCs. The impact of this dual temporal growth factor delivery on the local osteogenic differentiation and vessel formation was evaluated under static culture conditions. Figure 6A,B displays the distribution and intensity of red in alizarin red staining. The variation in red color intensity among groups reflects the extent of Ca^2+^ deposition within the scaffolds. Significant differences in red color intensity were observed between images taken on day 7 and day 14 for the BMP-2 treated group, as well as between day 7 and day 21 for the osteogenic medium group.

Assessment of ALP activity is a commonly used method for quantifying the osteogenic differentiation.^33,34^ ALP activity significantly increased in the capsule-incorporated group on both days 14 and 21 when compared to both osteogenic medium and no-induction groups (Figure 6C). This gradual increase in activity can be attributed to osteogenic differentiation initiated by the presence of BMP-2. These findings align with a study by Tian et al., which emphasized ALP as a vital marker for osteogenic differentiation, closely associated with the presence of BMP-2.^35^

In order to analyze the effects of spatial delivery of both growth factors BMP-2 and VEGF, samples from day 7 to day 21 underwent double immunostaining with antiosteopontin and anti-CD31. Immunofluorescence staining for osteopontin is frequently used in the evaluation of ASC osteogenic differentiation.^36^ The results demonstrated that spatial delivery of growth factors successfully induced local vascularization in the canal section and osteogenesis in the remaining regions of the scaffold (Figure 6D).

### 3.6 Perfusion Culture

**Figure 7:**
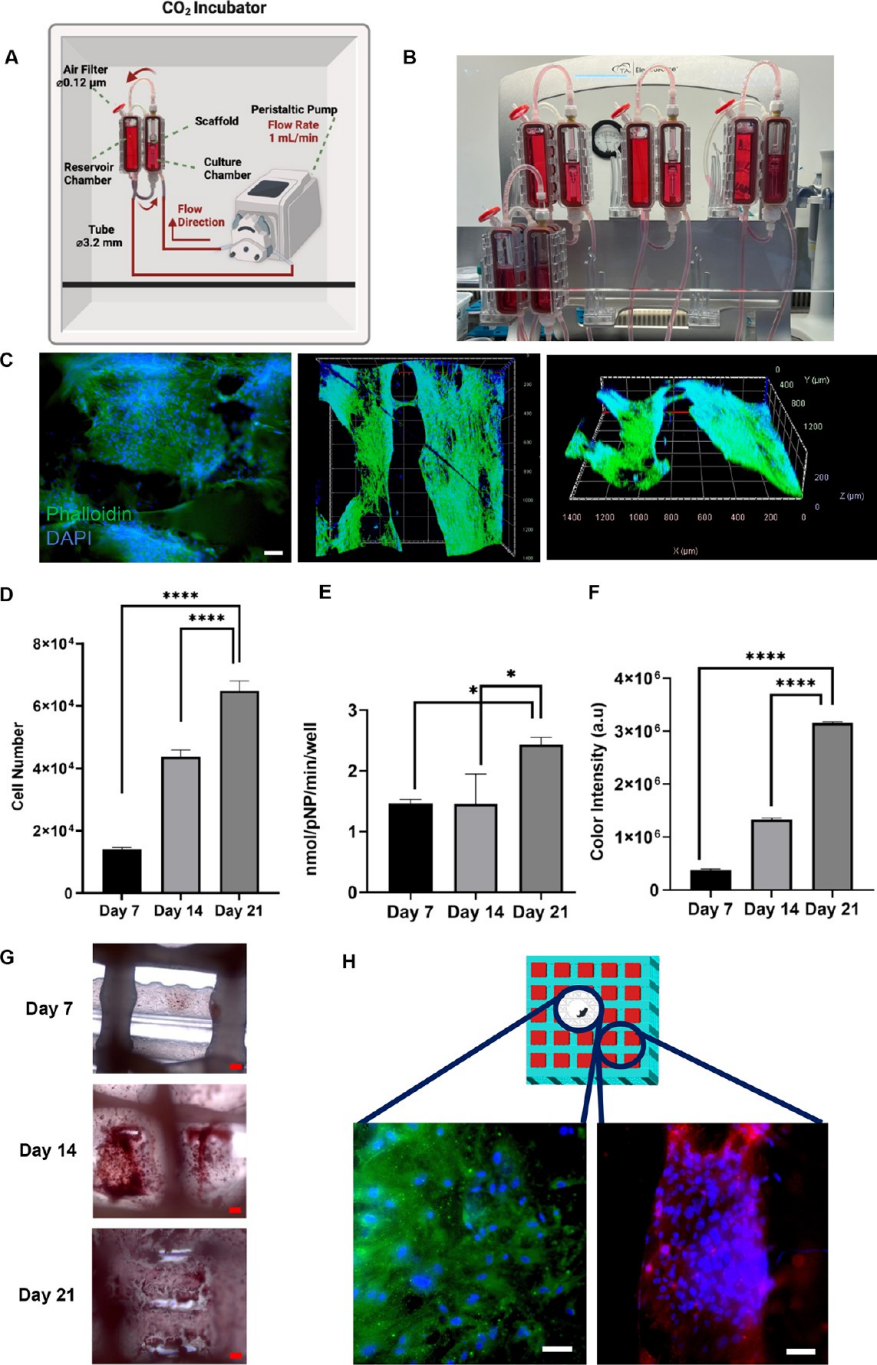
(A) Schematic representation of the perfusion bioreactor system that operates within the CO_2_ incubator, depicting the inlet, outlet, flow direction, and sample holders. (B) Assembly of the perfusion bioreactor system showing the connection ports and tubing. (C) Phalloidin (green)/DAPI (blue) staining of cells cultured within the grafts in perfusion culture representing homogeneous cell distribution over the filaments of the graft (scale bar = 200 μm). (D) Quantification of the viable cell number within the 3D grafts during perfusion culture. (E) ALP activity indicated ASC osteogenesis under perfusion culture during 21 days. ALP enzyme activity reflects the amount of *p*-NP formed per minute. Data is expressed as mean ± standard deviation of three samples. (F) Quantification of color intensity in alizarin red staining (*p* values: *<0.05, ****<0.0001). (G) Light microscopy images of the representing alizarin red staining of samples under perfusion culture for days 7, 14, and 21. Scale bar = 100 μm. (H) Immunofluorescent staining for osteopontin (red), CD-31 (green), and DAPI (blue) on day 21 under perfusion culture. Scale bar = 50 μm.

Scaffolds 3D bioprinted together with the spatial osteogenic and angiogenic bioinks were cultured under perfusion culture (Figure 7A,B). ASC- and dual growth factor delivery system-laden scaffolds were cultured with the bioreactor for 21 days. At the end of days 7, 14, and 21 in the perfusion culture, cells were fixed and stained for their actin filaments and nuclei to examine cell migration and morphology within the grafts (Figure 7C). The number of viable cells was determined by analyzing the images stained with DAPI, as shown in Figure 7D. It was observed that there was a statistically significant increase in the number of cells at day 21 of the perfusion culture.

ALP activity assessment was performed in order to quantitatively measure osteogenic differentiation. The amount of *p*-nitrophenyl phosphate formed in each chamber under dynamic culture per minute was read as the absorbance of *p*-nitrophenol (Figure 7E). It was observed that along with the number of cells, the amount of total ALP also increased, although it was not as statistically significantly different as compared to the cell number as the culture period increases.

The intensity of red color and the color distribution in the images of alizarin red-stained grafts cultured under perfusion culture reflect the calcium depositions on the sample due to osteogenic differentiation. Similar to the pattern observed in ALP activity, there was a notable increase in calcium (Ca^2+^) deposition over the course of 21 days. Importantly, this increase was statistically significant (*p* *<0.0001) when compared to the changes in ALP activity Figure 7F. The quantified (intensity of red color per image) color intensity results are presented in Figure 7G, which was correlated using the ImageJ tool. A white area was selected for background subtraction, and measurements for average light intensity were done from five different regions of interest with the same area.

To monitor osteogenic differentiation and vascularization within the graft simultaneously, 3D grafts were fixed and double-stained with antiosteopontin/anti-CD31 primary antibodies. It was observed that similar to the results observed under static culture, vascularization in the canal section and the osteogenesis in the rest of the scaffold were successfully induced by the spatial growth factor cues provided (Figure 7H).

**Figure 8:**
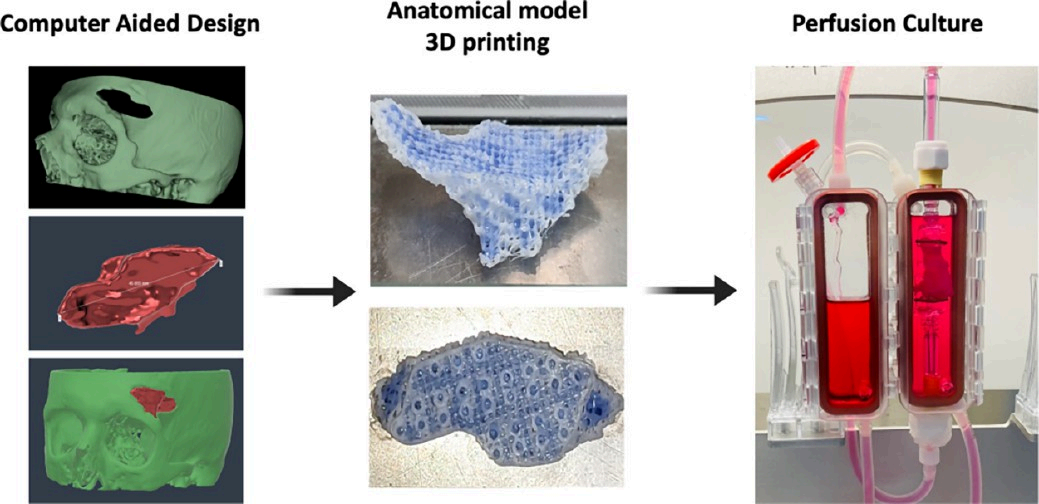
Patient DICOM data with cranial defects were obtained from MRI scans. The 3D model generation and implant design were performed using MIMICS software (Materialize) according to these defects (left-hand side). After radiological image segmentation, 3D image creation (surface rendering) was carried out, followed by 3D implant design using the MIMICS software’s “Materialise-3-matic” module. 3D models produced for patients from DICOM images were obtained as .stl files and further used in the 3D printing, as shown in the middle images. Anatomically shaped scaffolds were 3D printed with two straight-vessel configurations within the unit volume together. Moreover, these structures are 3D bioprinted with osteogenic and angiogenic bioinks in the bone and vessel regions to induce spatial ASC osteogenesis and induction of vascular structures. The anatomically shaped vascularized bone scaffolds can be cultured within a perfusion bioreactor system.

It was also shown as a proof of principle that anatomically shaped scaffolds can be produced with the two straight canal structures per unit volume configuration to produce prevascularized scaffolds that are coprinted with osteogenic and angiogenic bioinks. Additionally, it was shown as a proof of principle that the anatomically shaped grafts can be cultured within the perfusion bioreactor system, as shown in Figure 8.

## 4 Discussion

The tissue engineering field actively seeks solutions to meet the need for large-scale, vascularized bone substitutes for clinical transplantation. However, most of the strategies applied involve complex material combinations that are less feasible and harder to adapt in clinical applications. With this work, it was scoped to fabricate an off-the-shelf construct produced from simpler components (PCL as the only load-bearing component, alginate as the bioink material, and ASCs as the single source of cells) to produce a vascularized bone graft. The graft was designed and 3D printed to mimic the native macro- and microarchitecture of bone including the Haversian canal systems. In order to make the bone substitutes functional, osteogenic induction and control of cellular behavior within the engineered graft should be taken care of. If these parameters are controlled, it is possible to provide osteogenic differentiation of ASC and vascularization can be observed. BMP-2 and VEGF are two potent growth factors that stimulate osteogenesis and angiogenesis of ASC populations, respectively. In this work, the spatial delivery of BMP-2 and VEGF was applied by positioning capsules that encapsulate them within specific regions of the 3D-printed construct.

Higher amounts and relatively uniform capsular structures were produced by using a 10% w/v polymer solution. In the preliminary studies, it was found that 5% PCL concentration was not sufficient to form capsules; thus, the SEM characterization of that batch could not be conducted. The insufficient formation and particle loss during fabrication concur with the previous results.^4^ Particle size distribution for increasing concentrations of PCL is distinctively explained in Figure 2C. The average diameter of the 10% w/v batch is found to be 1200 nm, where the average goes up to 1900 nm for 15 and 20% w/v batches. Since Iqbal et al.^37^ report that average particle size is dependent on the duration of the ultrasonication process, the period of sonication can be optimized for obtaining better particle distribution. In further studies, new modification methods for the particle production process can be investigated to decrease the average particle size. Byun et al.^38^ suggested that an increased polymer concentration results in higher efficiency since the average particle size is higher. Since bigger particles lead to needle tip obstruction during 3D printing, it was determined to continue with 10% polymeric concentration rather than increasing the encapsulation efficiency.

In light of capsule diameter, EE, and release profile results, the most suitable polymer concentration was selected in order to be used in the controlled release of growth factors. Even though the EE% is higher in 15 and 20% w/v groups, total cumulative BSA release at the end of 21 days is found to be greater in the 10% PCL concentration; thus, the 10% PCL concentration was performed in VEGF and BMP-2 encapsulation.

Scaffold architecture was reported to have the utmost importance, in terms of cell response. It was found that SG-1 has the highest compressive modulus; however, due to its diminished porosity, it was not suitable for in vitro studies. That is why SG3′s scaffold geometry with a 1 mm fiber distance has the best trade-off in terms of porosity and Young modulus. Numerous studies conducted^31,39−41^ reported similar porosity and pore interconnectivity for the scaffolds.

In order to imitate the Haversian canal system in the bone and thus to create vascular structures, a central structure vessel design was carried out inside the square grid structure that represents the bone structure. When the Young’s modulus values are compared, it was observed that the Young’s modulus value decreased when TV or FV vessel structures were added per unit volume. When the situation in the unit volume of vessel placement is straight or diagonal in bilateral placement, it was determined that there is a decrease in cross-location. When the vessel structures in the unit volume are examined in the case of twisted compared to the straight layout, a high Young’s modulus value was obtained in the single slalom (SS). Lower and close values in the double (TS) and quadruple (FS) slaloms were obtained. When compared with all channel models except SS and TV, it is observed that the SV model gives significant differences in Young’s modulus value.

The cell viability and uniform distribution of cells across the grafts were critical indicators of construct success. Utilizing Live/Dead cell viability assays and Alamar Blue assays, we evaluated the effects of different experimental groups including those with and without encapsulated growth factors. Intriguingly, we observed that cell viability was well maintained within the alginate bioink during the fabrication process with cells promptly colonizing the scaffold surface by day 7. The incorporation of PCL carriers did not substantially alter the viable cell count, suggesting favorable compatibility of this composite system. Importantly, the induction of osteogenic differentiation yielded valuable insights with a noticeable reduction in cell proliferation compared to the control group. This finding underlines the influence of microenvironmental cues on cellular behavior, aligning with our previous findings and reports by other researchers.^31,42,43^ Furthermore, when comparing induction medium groups, the presence of BMP-2 and VEGF within capsules demonstrated minimal impact on cell viability, affirming the controlled and targeted delivery of these growth factors.

The success of our approach hinged on the effective delivery of BMP-2 and VEGF within the grafts, stimulating osteogenic and angiogenic processes. Alizarin red staining enabled the visualization of calcium deposition, which is a key indicator of osteogenic differentiation. The variation in red color intensity across time points reflected dynamic changes in calcium deposition, emphasizing the role of temporal growth factor cues. Furthermore, the quantification of ALP activity, a hallmark of osteogenic differentiation, highlighted the efficacy of the dual growth factor delivery system. This observation aligned well with previous studies that demonstrated the pivotal role of BMP-2 in driving osteogenic differentiation pathways through ALP expression.^44−46^ Immunofluorescence staining corroborated the successful local induction of osteogenesis and vascularization within the scaffold, underscoring the potential of our spatial growth factor delivery strategy.

Transitioning into perfusion culture allowed us to explore the effects of continuous nutrient supply and mechanical stimulation on graft development.^47^ The increase in cell number and ALP activity over the 21-day period hinted at the dynamic interplay between perfusion conditions and cellular behavior. Interestingly, the observed increase in calcium deposition, as indicated by Alizarin red staining, exhibited a statistically significant rise compared to ALP activity, indicating an intricate relationship between mineralization and osteogenic markers. The combination of dual staining with antiosteopontin and anti-CD31 antibodies further confirmed our scaffold’s ability to concurrently induce vascularization and osteogenic differentiation.

Moreover, our study provided a proof of concept for anatomically shaped, prevascularized grafts cofabricated with osteogenic and angiogenic bioinks. This advancement holds significant promise for personalized regenerative medicine strategies, where patient-specific constructs could be tailored to mimic native bone architecture and functionality.^48^

## 5 Conclusions

In this work, the spatial delivery of BMP-2 and VEGF was applied by positioning capsules that encapsulate them within specific regions of the 3D-bioprinted construct. Results of both static cell culture and perfusion cell culture studies showed that ASCs were successfully proliferated within the grafts. In addition to positive immunostaining for the osteogenic marker osteopontin, alizarin red staining and quantification of ALP production results quantitatively reflect that osteogenic induction of ASCs was achieved. Moreover, the vascularization process of the endothelial population within ASCs was evaluated with immunostaining for the CD31 marker, which also proved spatial vascularization through induction with VEGF. In conclusion, this work presents a novel yet simple vascularized bone graft production procedure that can possibly be developed to be an off-the-shelf product for clinical use in the future.

## Supporting Information Available

Bioprinted vascularized bone scaffolds cultured in a perfusion bioreactor system (3D CulturePro Electroforce, TA Instruments) for 21 days to ensure the effective nutrition and homogeneously distributed cell survival in the 3D matrix (MP4)
